# A Case for Open Network Health Systems: Systems as Networks in Public Mental Health

**DOI:** 10.15171/ijhpm.2017.01

**Published:** 2017-01-08

**Authors:** Michael Grant Rhodes, Marten W. de Vries

**Affiliations:** ^1^Mind Venture International, Maastricht, The Netherlands.; ^2^Department of Psychiatry and Psychology and CAPHRI Research School, Maastricht University, Maastricht, The Netherlands.

**Keywords:** Social and Economic Networks, Public Mental Health (PMH), Non-hierarchical Organization Governance, Adaptive Systems

## Abstract

Increases in incidents involving so-called confused persons have brought attention to the potential costs of recent changes to public mental health (PMH) services in the Netherlands. Decentralized under the (Community) Participation Act (2014), local governments must find resources to compensate for reduced central funding to such services or "innovate." But innovation, even when pressure for change is intense, is difficult. This perspective paper describes experience during and after an investigation into a particularly violent incident and murder. The aim was to provide recommendations to improve the functioning of local PMH services. The investigation concluded that no specific failure by an individual professional or service provider facility led to the murder. Instead, also as a result of the Participation Act that severed communication lines between individuals and organizations, information sharing failures were likely to have reduced system level capacity to identify risks. The methods and analytical frameworks employed to reach this conclusion, also lead to discussion as to the plausibility of an unconventional solution. If improving communication is the primary problem, non-hierarchical information, and organizational networks arise as possible and innovative system solutions.

The proposal for debate is that traditional "health system" definitions, literature and narratives, and operating assumptions in public (mental) health are ‘locked in’ constraining technical and organization innovations. If we view a "health system" as an adaptive system of economic and social "networks," it becomes clear that the current orthodox solution, the so-called integrated health system, typically results in a "centralized hierarchical" or "tree" network. An overlooked alternative that breaks out of the established policy narratives is the view of a ‘health systems’ as a non-hierarchical organizational structure or ‘Open Network.’ In turn, this opens new technological and organizational possibilities in seeking policy solutions, and suggests an alternative governance model of huge potential value in public health both locally and globally.

## Introduction

In December 2014, a 44-year-old man was killed in a violent incident at the Salvation Army in Maastricht, the Netherlands. The victim and perpetrator were known to the police, the local public health service-authorities (*Gemeentelijk Gezondheids Dienst [GGD]* and the public mental health (PMH) service the *Geestelijk Gezondheidszorg [OGGZ*]). The perpetrator was a classified “care-evader”; an increasing percentage of the 60 000 individuals that are estimated at high-risk of disruptive behavior and unresponsive to efforts to provide care and support.^1^

In the Netherlands, considerable efforts are made to actively monitor such persons, also through a PMH system. Incidents involving so-called confused persons (from Dutch) have also been increasing, just as public and mental health and community services, particularly those of *GGD* and *OGGZ,* have been under pressure to reform under a process of rapid decentralization.^2^

This paper presents observations from the initial investigation into the murder, and subsequent discussions to operationalize the investigation’s recommendations. These recommendations may also be relevant in the on-going international search for improvements and continuous progress in global public health.

## Background and Case

In the Netherlands, charitable organizations traditionally undertook the job of helping multi-problem individuals on the streets. Not until 2006, did a process of professionalization start under a National Plan including community services and mental health expertise.^3^ No sooner had the plan to integrate such services into the wider (public) health system emerged, than a period of fiscal retrenchment led to proposals for the activities to be decentralized under the 2014 ‘community, *Participation Law.’*^4^ Local governments were expected to find the funds to maintain the 2006 integrated system or “innovate.” It is left to local authorities what that innovation might be.

The murder therefore took place at a time of considerable national debate around the challenges of multi-problem individuals and the capacities of local government to manage. The extreme graphic violence of the incident raised media and political attention. The response of city authorities was to initiate a series of investigations. One such investigation, concerned the functioning of the public (mental) health authorities (*GGD* and *OGGZ*).^5^

The first finding of the original investigation was that the population affected was changing. Until a decade ago, visitors to civic organizations were primarily characterized by: alcohol, substance abuse, and unmanageable social problems. Research suggested that this growing population was also increasingly defined by mental health problems. Point prevalence rates ranged from 30% (The Hague) to 42% (Maastricht) to 60%-70% (Heerlen).^6-8^ Double to treble general population prevalence.^9^

The initial investigation concluded that the staff of the Salvation Army acted as best they could during the incident. Resource cuts as a result of the Participation Act could be identified, but the primary failure was one of communication in a fragmenting field of PMH providers. The *Participation Law* and the decentralization of funding obligations had led to increasing fragmentation, reducing collaboration, and increasing parallel operations resulting in PMH organizations taking defensive and competitive positions. This led to potentially disastrous mis- and/or non-communication over vital clinical case and social issues.

The initial investigation went on to suggest that if a failure of communication, and the potential for gaps in the safety net, lay at the heart of the initial problem; could innovations in communication and connection lie at the heart of any solution? Can a provider ‘network’ that is failing to connect and communicate be re-wired? The conceptual framework and network-based approach to health systems that informed the initial investigation is unique in that it clearly distinguishes between ‘supply’ and ‘demand’ social and economic networks in health systems.^10^ This makes it possible to narrow down on the function of specific ethnographic, social and economic characteristics of individual and organization networks or nodes and, the network functions and types these form.^11^ In turn, this helped formulate possible (re-)networking solutions.

In the background of this process, while generous central OGGZ funding of approximately €6 billion per annum continued, a perceived divide between those seeking and those providing services, has been a widely cited reasons for the system as a whole to seek a more innovative and sustainable approach to operations.^12,13^ The solutions and proposals evolving in Maastricht can be illustrated by first looking at the achievements and limitations of the 2006 National Plan.

## An Evolving Network-Based Approach to “the System”

The aim of the 2006 National Plan was to create a so-called (national) integrated public (mental) health system. This plan was extensive including both medical and social service providers, professional and (aspirant-) non-professional individuals and groups. The plan was therefore also compliant to a large body of public health literature in this area and a policy heavily debated in various European countries.^14^ Fragmentation was to be reduced and quality and efficiency increased. Under such an approach all operating entities become not only connected, but part of one single structure. The process is facilitated by public (ie, collective and compulsory) financing and subsequently provider funding.^15^ This “integrated health system” or ‘pyramid,’ forms the basis of much traditional public health planning, narratives and approaches in both the Netherlands and internationally.^16,17^

However, if we draw a network diagram of such a traditional ‘integrated public health system’ what is actually revealed is a centralized ‘hierarchical’ or ‘tree’ network. In such a network, although data for decision-making and control may be gathered from the branches, information dissemination and coordination flow from the top down, as does funding to specific organizations that function as ‘nodes’ in the network. Across this network: information and knowledge communication flows can be viewed as the ‘weak bonds’ of sociological/ethnographic networks, and; resource and contractual bonds as ‘strong bonds’ of economic networks. Viewed in these terms, the effect of the Dutch *Participation Law* was simply to reduce layers of coordination from the top down. In doing so, coordinating communication/information (weak) bonds were severed as economic and resource bonds were cut back. The hierarchical network, thus, succumbs to its principle weakness, failures resulting of de-capitation and the loss of the critical central network node(s).

We suggest, however, that cutting back on resources and even severing economic strong bonds does not necessarily mean that information flows and bonds must also severed.

**Figure 1:**
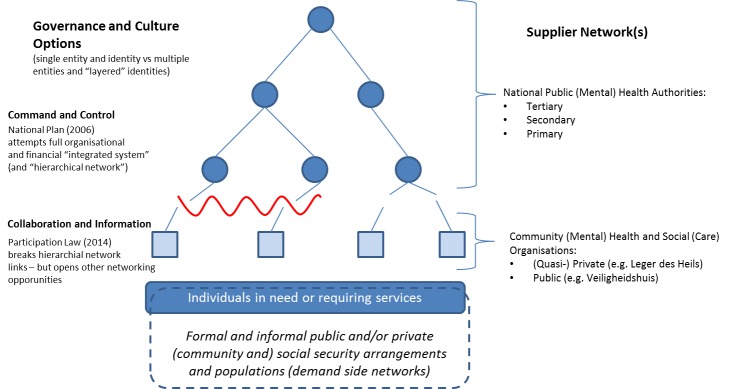
Re-integration Is not the Only Option: The PMH Integrated System Expands (2006) and Contracts (2014).

Figure 1 illustrates how the integration of social organizations and networks into the greatly enlarged “integrated system” or hierarchical network under the 2006 National Plan went into reverse. The analytic and policy response using established public health definitions of the health systems,^15^ left only one option; to attempt to reverse ‘fragmentation’ and rebuild what has been lost. The typical result, however, is simply a pruning of the ‘tree network,’ leaving the core structure and indeed the basic public health culture and internal narratives fundamentally unchanged. This ‘prune the tree to keep it healthy’ approach is evidenced in most recent country-wide and international proposals.^11^

On the other hand, as in the investigation reported here, if we change the framework of analysis from traditional ‘health systems analysis,’ to building and maintaining social, economic and subsequently communication (information and knowledge), ‘networks’ for PMH, more options become available.^12^ We can ask, “why prune a tree-, when you can catalyze the birth of a star-, network?” Severing resource bonds does not necessarily mean that information bonds must also severed. Furthermore, if improving communication and information sharing is the first order problem, non-hierarchical ie, distributed, star or meshed information networks arise as a clear alternative. The major challenge for this to be achieved is no longer information sharing, information technology has transformed this and created new possibilities. The major challenge remains to operationalize and create the human networks and a social-economic structure to enable this. Put simply, the problem is that the human and organizational networks involved have to have, or have to develop, individual ‘nodes’ in the network with shared purpose and uses for the information to be shared. We suggest that shared purpose can develop and function without shared management or direct control.

Unexpectedly, the ethnographic approach used in the early stages of the initial investigation started to evolve temporarily into just such an alternative network formation. Despite organizational fragmentation and competition, the investigation rekindled professional collaborations ‘within’ front-line organizations that had lain dormant for some time.

## Towards an Open Network Public Mental Health System

During the initial investigation, convergence amongst the previously fragmented PMH actors in Maastricht was achieved through a participative, group research setting. The approach used allowed diverse facets that contributed to any potential failures at the time of the murder to emerge, while obviating the untoward tendency of an exclusive focus on blame or decontextualized individual issues.^18,19^ A so-called Triade framework emerged that kept three essential aspects of the problem-solving discussion together: the problem itself (individual, situation, etc); the identifying parties and institutions (police, mental health institutions, Salvation Army, etc), and; the social context (neighborhood, social and community issues, etc).^6^ The ‘Triade’ was not a specific solution or intervention rather it was simply a *shared narrative* (words and stories) around which the diverse stakeholders could converge and move the process on to a more inclusive outcome.

A second point of convergence developed as individuals could seek personal and confidential consultation with the lead investigator (co-author and internationally recognized leader in the field of social-psychiatry). This was agreed within the group. Critically, when viewed as a network process, this added a physical, confidential but observable, connection between otherwise diverse individuals across fragmenting groups and organizations. From an ethnographic perspective, this also became a *shared symbol* of trust facilitating connectedness while maintaining distance and their respective organizational integrity and identity. As a result, the participants in the investigation started to develop multiple level or double identities such as the layering of employer/organizational-, and medical/professional-identities. The result was an informal social/medical/professional network within a wider and indeed diversifying network-field of community organizations. Subsequent debate focused on options to formalize such networking. The experience during the investigation also demonstrates that the role of a catalyst or person symbolic of convergence may be essential in the emergence of any horizontal open network.

**Figure 2:**
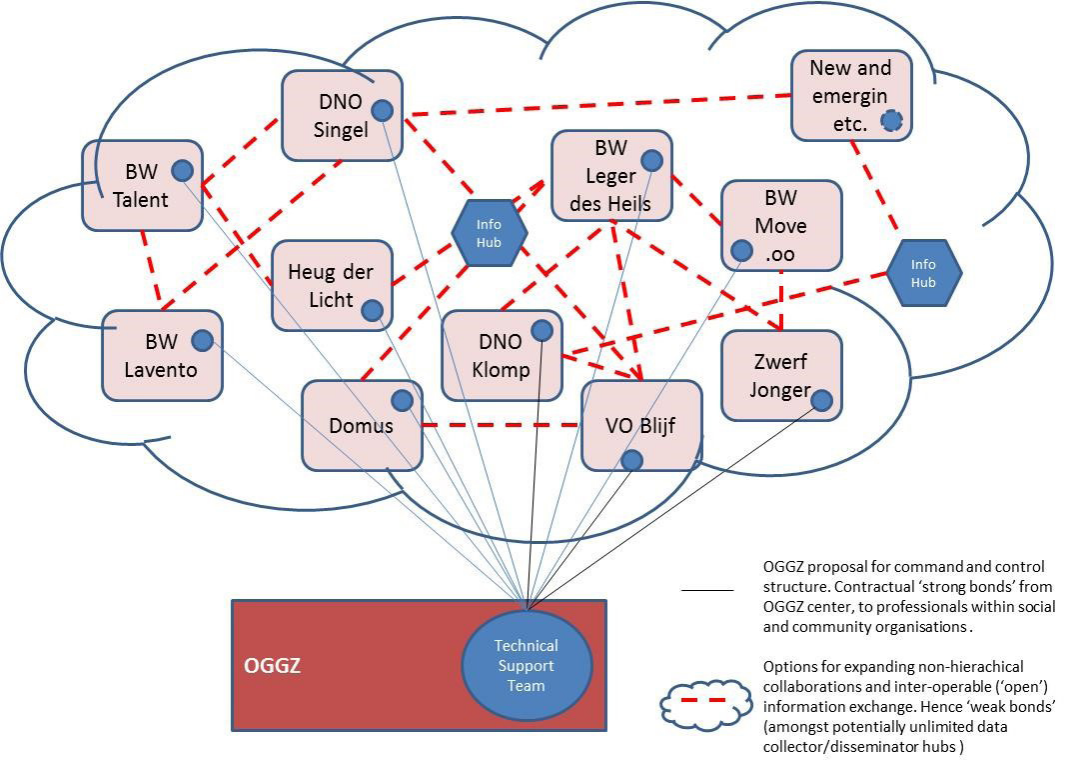
Two Perspectives on the Evolving ‘Network’ of the Public Mental Health (PMH) ‘System’ in Maastricht, the Netherlands.

The first result was an internal OGGZ proposal to formalize the emerging network of hitherto fragmented front-line services through a centrally resourced (ie, ‘strong bond’) network of key staff positions from each institution involved in community and mental health services (Figure 2).^20^ ‘Strong’ contractual bonds then overlay ‘weak’ information sharing bonds between these professionals. But since such cooperation requires the formal agreement of the directors of the social and community organizations, the process illuminates a further level of potential cooperation and an alternative network evolution amongst those devolved organizations.

Most traditional pre-2006 OGGZ/PMH organizations survived decentralization and remained publicly funded in a hierarchical network of institutions. For the OGGZ, the move to extend the internal hierarchical organization to community level and place professionals within a growing field of external and predominantly social and community organizations can be seen as a way of ensuring some control and coherence in mental health services and quality. The policy frame of ‘control’ of an ‘integrated’ ‘public (mental) health system’ could be secured.

The Participation Law gave rise, however, to increasingly differentiated social and community organization in terms of: organizational identities and values (Christian, Socialist, etc); operating foundations and legal bases (eg, commercial, voluntary, mixed [‘social enterprises’], etc), and scope (psycho-social care, housing, food, well-being programs, economic activities, social activities, etc). The differentiated organizational identities are then central to their respective ‘business models’ (including mobilizing non-financial production factors such as volunteer time and other resource contributions). The new “partner,” or demand-side agents and networks of these organizations were also diversifying in response to having been limited to public funding agencies under the 2006 National Plan. The first proposal for a ‘closed’ network of PMH professionals could therefore only exist because of a willingness amongst the diverse organizations to find areas of collaboration.

The initial murder investigation created a second result and proposal. The one area of ‘collaboration amongst competitors’ identified was information and knowledge sharing; particularly with regard to a growing number of potentially “confused persons.” How might this willingness be built upon? One approach is to create inter-operability of internal information systems, for example the sharing of client service use information by each authorized organization. The usually invisible world of non-hierarchical organization and professional information and knowledge sharing is revealed and can be shared in solid metrics of bits and bytes. At the same time, open data of this sort implies creating the possibility of any number of data hubs or centers. The OGGZ can be one such hub or ‘boundary spanner.’ The lost national-community wide mental health network is re-built on the foundation of purely ‘weak bonds’ ie, information sharing. As in Figure 2, hybrids of hierarchical and non-hierarchical networks may also develop. The communication gap identified in the initial investigation is thereby closed.

The longer-term answer would therefore depend on whether collaboration can be made to work for the mutual benefit of all contributing and participating actors. The importance of anticipating the requirements and maintaining the trust, of potential service users – ie, those whose data is ultimately to be shared – should also not be under-estimated. The second option we put on the table is therefore to explore the potential for a much wider, non-hierarchical organization or ‘open network’ of mental health-related services providers build on a foundation of the ‘weak bonds’ of information and knowledge sharing and harmonization.

## Conclusion: How and Where to Start?

During a period of healthcare planning flux and painful restructuring, no event, however emotive, is likely to be spared the primary reflex of the policy-maker; never waste a good crisis. But, the case presented illustrates two potentially important lessons. Lessons we hope will provoke debate. First, the narrative and existing definitions of ‘integrated health systems’ has “locked-in” public health and PMH to policy or ‘system standard’ approaches entirely focused on creating and maintaining ‘hierarchical networks.’^21^ If we can overcome the transaction costs of breaking out from established narratives and approaches, and view ‘health systems’ as parts of interacting ‘networks,’ other front-line diversified and operationally sustainable possibilities emerge.

Secondly, non-hierarchical networks (ie, distributed, star or meshed networks) are possible in health and social (-psychiatric) services as much as they are in computer networks; but they require the development of double- even multi-level identities at individual and organizational levels. In turn, these identities provide or articulate the bonds that can forge micro-, meso- and ultimately macro-networks of networks. Managing, leading and/or facilitating such non-hierarchical organization is unlikely to be straight-forward; but it is possible and not uncommon.^22^ On the other hand, the case also illustrates the easily underestimated challenges, of achieving and maintaining expansionary “integrated health systems.” But comprehensive integration does remove the need for more detailed or subtle ‘ethnographic’ understanding and engagement, particularly in sustaining interaction with demand-side issues and networks essential to the workings of an open network approach.

The final lesson of the case may only reveal itself in time. The catalyst for self-organization is unlikely to be found solely amongst motivated and concerned professional service providers and networks. The murdered man, a retired boxer and reforming drug addict, was himself a volunteer trying to help those like his eventual killer navigate the increasingly arcane world of psycho-social support services available after 2006. Elsewhere in the Netherlands, initiatives are afoot to empower such individuals with on-demand information on the array of available service in real time; *AirBnB* for the homeless.^23^ This is a demand that if it can be satisfied, could potentially reduce conflict and unhappiness, if not save lives. An ‘open’ and or ‘collaborative’ network, or hence ‘movement’ as much as ‘organization,’ that prevails would seem likely to be one where supply meets such demands.

## Acknowledgements

We thank: Prof. Luc Soete for his comments on early drafts of the paper; the staff of the Salvation Army, OGGZ institutes and the City of Maastricht, the Netherlands for their cooperation in the research and subsequent organizational development; the anonymous reviewers for their diligence and; Walid Ammar (MOPH Lebanon) for the exchange of ideas relating local to international contexts.

## Ethical issues

Not applicable.

## Competing interests

The case reported is a self-funded activity on the basis of an initial investigation funded by the Municipal Government of Maastricht, The Netherlands. No conflict of interest.

## Authors’ contributions

Both authors contributed equally to the writing of this paper.

## Authors’ affiliations

^1^Mind Venture International, Maastricht, The Netherlands. ^2^Department of Psychiatry and Psychology and CAPHRI Research School, Maastricht University, Maastricht, The Netherlands.
